# Supplementary material for: Splice-Junction-Based Mapping of Alternative Isoforms in the Human Proteome
Source: Cell Rep. Author manuscript; Available in PMC 2020 Jan 15. (PMC6961840; doi:10.1016/j.celrep.2019.11.026)
Supplement: 3 [file NIHMS1546469-supplement-3.zip › DF2/PXD009737/TestisTrypsin-286-Q92823-LEVPLDLVQPPTITQQSPK.pdf]

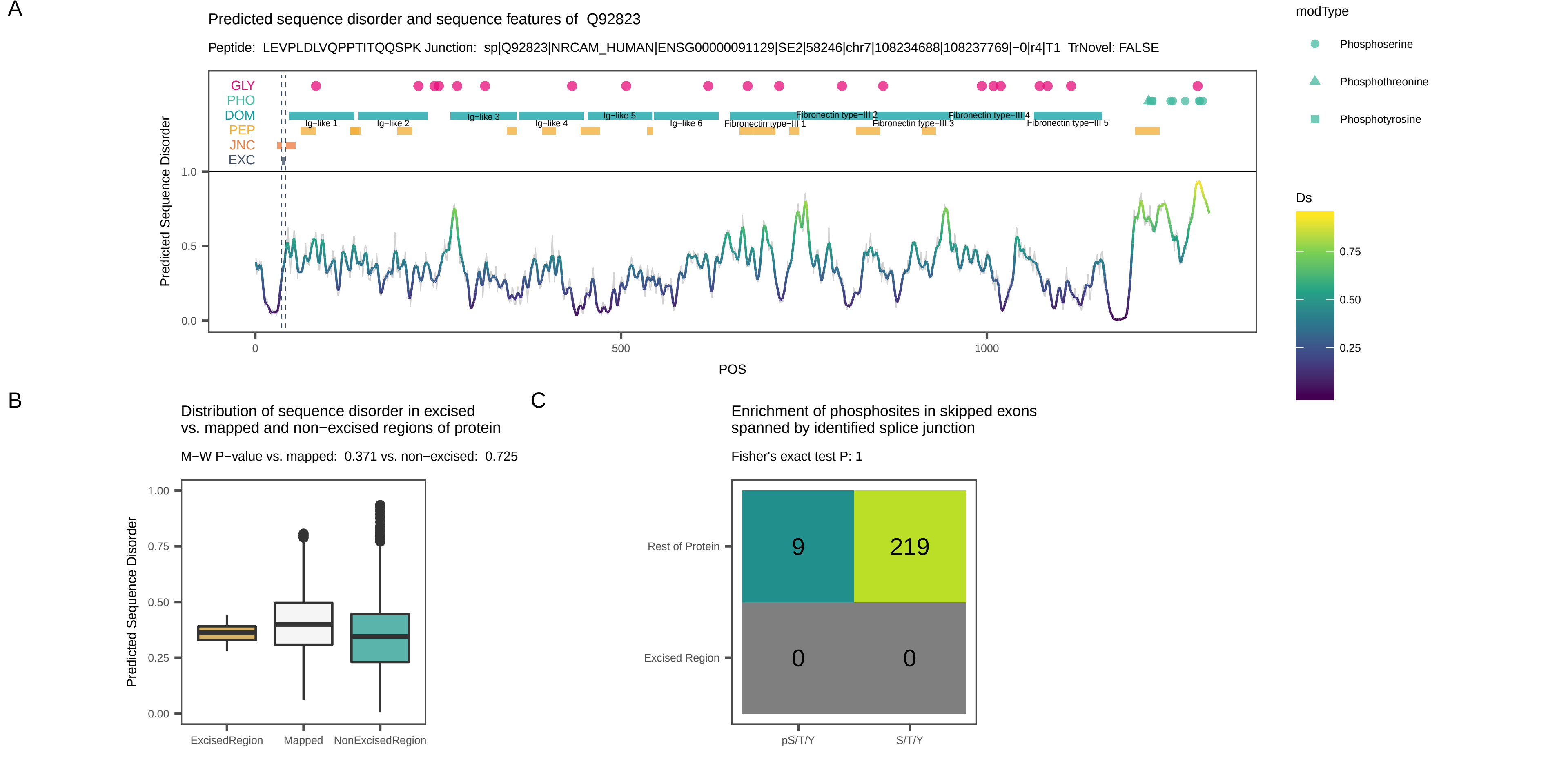
A
Predicted sequence disorder and sequence features of Q92823
Peptide: LEVPLDLVQPPTITQQSPK Junction: sp|Q92823|NRCAM_HUMAN|ENSG00000091129|SE2|58246|chr7|108234688|108237769|−0|r4|T1 TrNovel: FALSE
GLY
PHO
DOM
PEP
JNC
EXC
Ig−like 1
Ig−like 2
Ig−like 3
Ig−like 4
Ig−like 5
Ig−like 6
Fibronectin type−III 1
Fibronectin type−III 2
Fibronectin type−III 3
Fibronectin type−III 4
Fibronectin type−III 5
Predicted Sequence Disorder
1.0
0.5
0.0
0
500
1000
POS
modType
Phosphoserine
Phosphothreonine
Phosphotyrosine
Ds
0.75
0.50
0.25
B
Distribution of sequence disorder in excised vs. mapped and non−excised regions of protein
M−W P−value vs. mapped: 0.371 vs. non−excised: 0.725
Predicted Sequence Disorder
1.00
0.75
0.50
0.25
0.00
ExcisedRegion
Mapped
NonExcisedRegion
C
Enrichment of phosphosites in skipped exons spanned by identified splice junction
Fisher's exact test P: 1
Rest of Protein
Excised Region
9
219
0
0
pS/T/Y
S/T/Y
